# Section II.—Report on Dental Education

**Published:** 1897-04

**Authors:** Louis Ottofy


### Section I I.—Report on Dental Education.

BY LOUIS OTTOFY, D. D. S.

Presented to the American Dental Association.

Mr. President and Gentlemen: The Section of Dental Education, Literature and Nomenclature begs leave to offer the following as its annual report.

1ST. EDUCATION.

Since 1887 this section has regularly reported the number of dental colleges in active operation in the United States, with such additional information as has been deemed proper. The rule followed, simply aimed at a truthful report of the number of colleges, newly organized institutions, and such other incorporated bodies, as confer the dental degree. No attempt to discriminate, has been made. In-as-much as the list thus published, by this Association each year, is being accepted and offered as evidence of the existance of dental institutions, it seems manifestly improper, that institutions reputed to exist, but regarding which no information can be obtained, shall be placed side by side with institutions which are established and known to be engaged in the works of dental education.

This innovation, therefore, begins this year, of separating the dental schools of this country, according to the information in possession of this section regarding them, into three classes:

1. Dental colleges in active operation.
2. Dental colleges organized during the past year.
3. Corporations conferring the dental degree, but regarding which no further information is at hand. The following is the number of Matriculants, Students in attendance, and Graduates, as reported by each school for the session 1895-6 :

| | Matriculants. | Attendance. | Graduates. |
|---|---|---|---|
| ALABAMA.—*Birmingham Dental College, Birmingham | 36 | 36 | 6 |
| CALIFORNIA.—*University of California, College of Dentistry, San Francisco. | 195 | 195 | 52 |
| COLORADO.—*University of Denver, Dental Department, Denver | 31 | 28 | 8 |
| DISTRICT OF COLUMBIA.—*Columbian University, Dental Department, Washington | 63 | 56 | 14 |
| *National University, Dental Department, Washington | 47 | 47 | 6 |
| *Howard University, Dental Department, Washington | 13 | 12 | 3 |
| GEORGIA.—*Atlantic Dental College, Atlantic | 202 | 200 | 40 |
| *Department of Dentistry, Southern Medical College, Atlantic | 52 | 49 | 5 |
| ILLINOIS.—*Chicago College of Dental Surgery, Chicago | 436 | 399 | 107 |
| *Columbian Dental College, Chicago | 63 | 63 | 25 |
| Northwestern College of Dental Surgery, Chicago | 12 | 10 | 3 |
| *Northwestern University Dental School, Chicago, and American College of Dental Surgery, consolidated. | 548 | 543 | 149 |
| INDIANA.—*Indiana Dental College, Indianapolis | 157‡ | 157 | 43 |
| IOWA.—*Dental Department, State University of Iowa, Iowa City | 212 | 212 | 33 |

*Including one post-graduate.
†Including seven special students.
‡Including three special students.
§Including one special student.
‖ Application for membership favorably reported upon, announce that they will conform to the rules of the National Association of Dental Faculties.

| | | | |
|---|---|---|---|
| Kentucky.—*Louisville College of Dentistry, Louisville | 141 | 141 | 33 |
| Maryland.—*Baltimore College of Dental Surgery, Baltimore | 207 | 207 | 44 |
| †Baltimore Medical College, Dental Department, Baltimore | 34 | 30 | 3 |
| *University of Maryland, Dental Department, Baltimore | 204 | 202 | 42 |
| Massachusetts.—*Boston Dental College, Boston | 189 | 189 | 37 |
| *Dental Department of Harvard University, Boston | 104 | 103 | 22 |
| Michigan.—*Detroit College of Medicine, Department of Dental Surgery Detroit | 70 | 70 | 19 |
| *University of Michigan, College of Dental Surgery, Ann Arbor | 190 | 189 | 60 |
| Minnesota.—*University of Minnesota, College of Dental Surgery, Minneapolis | 90 | 84 | 14 |
| Missouri.—*Dental Department, Marion Sims College of Medicine, St. Louis. | 44 | 38 | 6 |
| *Kansas City Dental College, Kansas City | 149 | 135 | 47 |
| *Missouri Dental College, St. Louis | 101 | 96 | 29 |
| *Western Dental College, Kansas City. | 220 | 202 | 46 |
| Nebraska.—*Dental Department, University of Omaha, Omaha | ....... | ....... | ....... |
| New York.—New York Dental School, New Yoık | 16 | 16 | 3 |
| *New York College of Dentistry, New York | 339 | 309 | 74 |
| *Dental Department, University of Buffalo, Buffalo | 187 | 187 | 36 |
| Ohio.—*Cincinnati College of Dental Surgery, Cincinnati | 33 | 33 | 8 |

| | | | |
|---|---|---|---|
| *Cleveland University of Medicine and Surgery, Dental Department, Cleveland | 23 | 23 | 6 |
| *Ohio College of Dental Surgery, Cincinnati | 213 | 210 | 47 |
| *Ohio Medical University, Department of Dentistry, Columbus | 62 | 62 | 13 |
| *Western Reserve University, College of Dentistry, Cleveland | 53 | 53 | 7 |
| PENNSYLVANIA.—*Pennsylvania College of Dental Surgery, Philadelphia | 343 | 319 | 96 |
| *Philadelphia Dental College, Philadelphia | 409 | 400 | 116 |
| *University of Pennsylvania, Department of Dentistry, Philadelphia | 325 | 310 | 74 |
| TENNESSEE.—*Central Tennessee College, Meharry Dental Department, Nashville | 18 | 18 | 3 |
| *Tennessee Medical College, Department of Dentistry, Knoxville | 18 | 18 | 2 |
| *University of Tennessee, Dental Department, Nashville | 54 | 50 | 11 |
| *Vanderbilt University, Department of Dentistry Nashville | 154 | 150 | 29 |
| VIRGINIA.—*University College of Medicine, Department of Dentistry, Richmond | 36 | 36 | 13 |
| WASHINGTON.—*Tacoma College of Dental Surgery, Tacoma | 26 | 25 | 3 |
| WISCONSIN.—*Milwaukee Medical College and School of Dentistry, Milwaukee. | 65 | 60 | 6 |
| Total Members of the National Association of Dental Faculties | 6293 | 6013 | 1448 |

NEW SCHOOLS ORGANIZED DURING THE YEAR (NO SESSIONS HELD YET.)

COLORADO.—Colorado School of Surgery, Denver.

ILLINOIS.—International Dental College, Chicago.

PENNSYLVANIA.—Pittsburg Dental College, Department Western University of Pennsylvania, Pittsburg.

CORPORATIONS CONFERRING THE DENTAL DEGREE (NO OTHER INFORMATION AT HAND.)

ILLINOIS.—German-American Dental College, Chicago.

MISSOURI.—Kansas City College of Dental Surgery, Kansas City.

WISCONSIN.—Wisconsin College of Dentistry, Milwaukee.

TOTAL.

1. Dental Colleges in active operation........................ 46
2. Organized during the past year.......................... 3
3. Corporations conferring the dental degree................ 3

According to these statistics there were matriculated as students of dentistry during the past year 6,293 persons, 6,013 of whom were in actual attendance, and upon 1,446 of whom the dental degree was conferred.

The following table will indicate the increase in the number of graduates since 1886.

| | For the year. | Grand total. | | For the year. | Grand total. |
|---|---|---|---|---|---|
| 1886 | 503 | 1100 | 1892 | 1483 | 6708 |
| 1887 | 597 | 1846 | 1893 | 379 | 7613 |
| 1888 | 746 | 2642 | 1894 | 905 | 8821 |
| 1889 | 796 | 3605 | 1895 | 1208 | 10267 |
| 1890 | 963 | 4846 | 1896 | 1446 | |
| 1891 | 1241 | 6326 | | | |

*It is deserving of mention that every college now actively engaged in teaching, or which has been organized during the year, (except one whose announcement has not yet been published), is either a member of the "National Association" of Dental Faculties or an application is pending, or it has announced its intention to conform to the rules of the "National Association" of Dental Faculties.

In the later class there are but three colleges in the country and those intend to apply for membership, as soon as they have complied with certain necessary requirements.

This information might not be considered of much consequence, were it not for the fact, that the National Association of Dental Faculties, has at its session this week, taken one of the

*The decrease in 1893 and 1894 was due to the operation of the three course.

most important steps, in the history of Dental Education, by the adoption of a plan for the admission of students to colleges. Hitherto an applicant for admission presented himself for admission, and it was left wholly to the judgment of some official of the college to accept or reject. There is no doubt that this imperfect system resulted in the admission of many who were not properly qualified. By the plan just adopted, the requirements for admission into any of the colleges is identical, and the requirements are graded from year to year, in such a manner that beginning with next year the educational standing of our students will be higher and increase within four years to a plan practically equivolent to a High School education.

The Section deems this action one of the most important advances made in Dental education.

The Section has no further report to make on Dental Education or Literature, desiring these subjects passed without discussion, in view of the amount of time devoted to them last year.

